# “Farewell to the God of Plague”: The Importance of Political Commitment Towards the Elimination of Schistosomiasis †

**DOI:** 10.3390/tropicalmed3040108

**Published:** 2018-10-03

**Authors:** Jin Chen, Jing Xu, Robert Bergquist, Shi-Zhu Li, Xiao-Nong Zhou

**Affiliations:** 1 National Institute of Parasitic Diseases, Chinese Center for Disease Control and Prevention; Chinese Center for Tropical Diseases Research; WHO Collaborating Centre for Tropical Diseases; National Center for International Research on Tropical Diseases; Key Laboratory of Parasite and Vector Biology, Ministry of Health, Shanghai 200025, China; jinchen@nipd.chinacdc.cn (J.C.); xujing@nipd.chinacdc.cn (J.X.); stoneli1130@126.com (S-Z.L.); 2 Ingerod, SE-454 94 Brastad, Sweden; robert.bergquist@outlook.com

Schistosomiasis control in China has always been conducted with strong political leadership and support at the highest level of government [1]. In the 1950s, massive surveys were conducted. It eventually became clear that a large part of the country was highly endemic for schistosomiasis japonica. An estimated 11 million people were infected, and local infection rates ranged from 10 to 90% [1,2,3,4]. Realizing the severity of the situation in as many as 12 provinces along the Yangtze River, Chairman Mao Zedong himself took the reins and launched a call for schistosomiasis to be wiped out.

The Chinese Central Government prioritized control of the disease. Regarding it as a political issue, the mechanism of “political leadership, government support and mass involvement” was established in 1956. The Yujiang County in Jiangxi Province responded rapidly and worked out a multifaceted scheme with the focus on snail control through environmental modification, repeated screening and molluscicide treatment [5,6]. After the implementation of this strategy for two years, the county authorities reported that schistosomiasis had been eliminated in their area. Chairman Mao was jubilant. He would later spoke of jotting down “…as sunlight falls on my window, I look towards the distant southern sky and in my happiness pen the following lines”. What he referred to is the now famous, sonnet-style poem in two parts *Farewell to the God of Plague* that was published in the Chinese newspaper *People’s Daily* on 3 October 1958. (Figure 1) The poem eulogizes the remarkable achievement in Yujiang and encourages work towards schistosomiasis elimination in more endemic areas [5,6]. Inspired by these enthusiastic lines, active people in all endemic counties devoted themselves to schistosomiasis control following what became known as the Yujiang Model.

The year 2018 marks the 60th anniversary of this first step on the road that eventually should lead to the reality of schistosomiasis elimination. This goal has attracted increased support from the Chinese Government, promoting national schistosomiasis epidemiology surveys as well as research, control and prevention. However, political commitment as well as work at the bench and in the field have gone through different stages over the years. After Chairman Mao’s passionate approach in 1958, Chairman Jiang Zemin stated 45 years later that the control and elimination of schistosomiasis is the responsibility of the government. This spoken and written declaration promoted schistosomiasis control to a level of priority within the broad range of public health issues. Human, material and financial resources were allocated to the cause and the health sector was strengthened, which eventually led to intersectoral collaboration between several departments of the central government. This proved essential in the fight against the neglected tropical diseases (NTDs), schistosomiasis in particular. At the first stage (1950s–1985), the mass campaigns and vertical control was conducted with an emphasis on snail eradication. This was achieved through environment modification and molluscicide treatment of the snail habitats, together with auxiliary individual protection of the human population. As a result, the snail habitat were reduced by 11 billion m^2^ by 1981. Whole provinces met the criteria of schistosomiasis elimination: Guangdong Province and Shanghai Municipality in 1985, Fujian Province in 1987 and Guangxi Zhuang Autonomous Region in 1989 [6]. At the second stage (1986–2003), the chemotherapy-based morbidity control strategy with praziquantel treatment was prioritized through the World Bank Loan Project on schistosomiasis control 1992–2001 [3]. The number of cases and morbidity of the infected residents in the endemic provinces decreased by half [7,8]. During the third stage (2004–2015), the Chinese Government promulgated the Schistosomiasis Prevention and Control Regulation, which accelerated the implementation of an integrated strategy, focusing on controlling all sources of transmission [9,10]. In parallel with continued snail control, the interventions included agricultural mechanization (exchange of water buffaloes for tractors), the construction of lavatories with running water, and the institution of drug treatment and health education [11]. By 2015, all endemic provinces/autonomous regions had achieved control of the infection, i.e., the estimated number of infected people had fallen by a dramatic 91% (from 842,500 to 77,200) [12].

With respect to schistosomiasis, China is now saying ‘enough is enough’—this disease must go! According to the latest endemicity report in 2016, five out of 12 endemic provinces have eliminated schistosomiasis; one has reached the stage of transmission control, and six have met the criteria of transmission interruption [13]. Based on the achievement gained, the Chinese Government has issued a decree that schistosomiasis should be eliminated by 2025 [14], which would coincide with reaching United Nations’ Sustainable Development Goal: *End the NTDs* [15]. The Healthy China 2030 Plan requests that all counties still endemic for schistosomiasis reach the elimination stage via an integrated strategy and an efficient surveillance and response approach [16,17]. To achieve this ambitious goal, China must interrupt the transmission of schistosomiasis in more than 90% of endemic counties and eliminate the infection in 75% of them by 2020. They must also further achieve transmission interruption in all endemic counties and elimination in 90% of them by 2025 [13].

In parallel with the elimination of schistosomiasis at home, China also assists other endemic countries. This is based on the successful experience mentioned above and then tailored to local settings [18]. The China-Zanzibar-WHO project on schistosomiasis control is a good example of this strategy, adopting the model of high-level governmental communication and technical cooperation. At the 2014 World Health Assembly (WHA) in Geneva, Switzerland, the Government of Zanzibar, the World Health Organization (WHO) and China agreed to jointly work towards the control of schistosomiasis in Zanzibar. With financial support for advisors, health education of local staff and assistance with laboratory renovation from the Chinese Government and China’s Ministry of Commerce, a baseline survey and disease control study of urogenital schistosomiasis (due to *Schistosoma haematobium*) was initiated on Pemba Island in 2016. So far, the pilot intervention has completed a baseline survey providing current prevalence rates and the biological situation with regard to the local intermediate snail host *Bulinus globosus*. By systematic disease investigation and snail surveys followed by treatment in cooperation with the local NTD office, the prevalence of schistosomiasis haematobia in Pemba has already showed signs of reduction.

Schistosomiasis elimination in China has a 60-year history. Its early success needed Chairman Mao Zedong’s initiative and is now being advanced by long-term political commitment, along with strong developments in society and the economy. Repeating this achievement abroad will only be possible with long-term support from local governments and the incorporation of social and economic approaches. Local import and appreciation of Chinese technologies and products, such as the current Belt and Road Initiative project aimed at boosting local economies with global health development, will be crucially depended on to achieve the ultimate goal of worldwide elimination of the NTDs, including schistosomiasis.

## Figures and Tables

**Figure 1:**
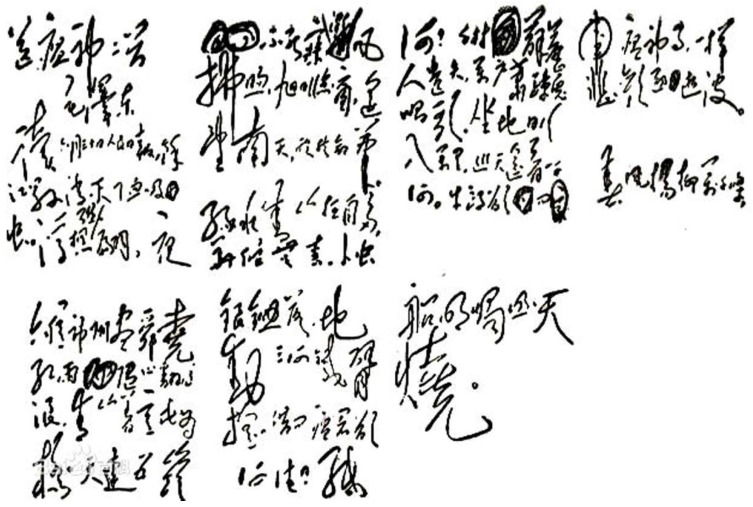
Chairman Mao Zedong’s “Farewell to the God of Plague" in his own handwriting.
